# Psilocybin microdosers demonstrate greater observed improvements in mood and mental health at one month relative to non-microdosing controls

**DOI:** 10.1038/s41598-022-14512-3

**Published:** 2022-06-30

**Authors:** Joseph M. Rootman, Maggie Kiraga, Pamela Kryskow, Kalin Harvey, Paul Stamets, Eesmyal Santos-Brault, Kim P. C. Kuypers, Zach Walsh

**Affiliations:** 1 Department of Psychology, University of British Columbia, Kelowna, BC Canada; 2 Quantified Citizen Technologies Inc., Vancouver, BC Canada; 3 Department of Family Medicine, University of British Columbia, Vancouver, BC Canada; 4 Fungi Perfecti, LLC, MycoMedica Life Sciences, Olympia, WA USA; 5 Department of Neuropsychology and Psychopharmacology, Faculty of Psychology and Neuroscience, Maastricht University, Maastricht, The Netherlands

**Keywords:** Psychology, Human behaviour, Drug development, Cognitive neuroscience, Learning and memory

## Abstract

Psilocybin microdosing involves repeated self-administration of mushrooms containing psilocybin at doses small enough to not impact regular functioning. Microdose practices are diverse and include combining psilocybin with substances such as lion’s mane mushrooms (Hericium erinaceus; HE) and niacin (vitamin-B3). Public uptake of microdosing has outpaced evidence, mandating further prospective research. Using a naturalistic, observational design, we followed psilocybin microdosers (*n* = 953) and non-microdosing comparators (*n* = 180) for approximately 30 days and identified small- to medium-sized improvements in mood and mental health that were generally consistent across gender, age and presence of mental health concerns, as we all as improvements in psychomotor performance that were specific to older adults. Supplementary analyses indicated that combining psilocybin with HE and B3 did not impact changes in mood and mental health. However, among older microdosers combining psilocybin, HE and B3 was associated with psychomotor improvements relative to psilocybin alone and psilocybin and HE. Our findings of mood and mental health improvements associated with psilocybin microdosing add to previous studies of psychedelic microdosing by using a comparator group and by examining the consistency of effects across age, gender, and mental health. Findings regarding the combination of psilocybin, HE and B3 are novel and highlight the need for further research to confirm and elucidate these apparent effects.

## Introduction

The use of fungi containing psilocybin to enhance health and well-being has a long history across diverse cultures^1^. After centuries of aggressive colonial suppression most recently manifested in the US-led “war on drugs”, psilocybin has reemerged outside of its traditional indigenous contexts, as a therapeutic agent to treat mental illness and enhance well-being. Indeed, discussions of the medicinal properties of psilocybin mushrooms have proliferated in mainstream North American and European culture in recent years^2,3^. This interest has focused predominantly on doses sufficient to engender dramatic alterations in consciousness; however, the use of smaller *“microdoses”* has also become a topic of substantial public and scientific interest^4,5^.

Microdosing involves regular self-administration of psychedelic substances in doses small enough to not impair normal cognitive functioning^6^. The most widely reported substances used for microdosing are psilocybin mushrooms and LSD, and to a lesser degree other psychedelic substances such as mescaline and 2-CB^7^. Surveys of microdosing psilocybin have identified diverse practices but generally converge on the self-administration, 3–5 times per week, of 0.1 to 0.3 g of dried mushrooms^7–12^. Improvements in mood, emotional well-being and cognition have been reported among the top motivations for microdosing^13^, and several cross-sectional studies have identified associations between microdosing and perceived improvements in mood^13–17^ and cognitive functioning^10,11,16^, reductions in stress^7^, depression^7,9,16^ and anxiety^7,9,14,18^.

Relatively few prospective studies have evaluated microdosing. The first longitudinal study of microdosing analyzed daily assessments of 98 microdosers for 6 weeks, and found acute transient improvements across broad domains of psychological functioning on microdosing days, and reductions in stress, depression and distractibility from baseline to study conclusion. Further, although that study’s conclusions are limited by the lack of a non-microdosing control group, supplementary examinations concluded that the observed effects were not consistent with what might be anticipated based on common expectancies related to microdosing^7^. A subsequent prospective study that followed 81 microdosers for four weeks also reported improvements across several domains of psychological well-being, including enhanced emotional stability and decreased anxiety and depression. However, supplementary analyses suggested that these positive effects may be attributable to expectancies and highlighted the need for further research with non-microdosing control participants to better distinguish the effects of microdosing from placebo effects and other longitudinal confounds^18^.

The effective use of placebo has presented a challenge in the few published studies that have attempted such a design in the context of psychedelic microdosing^17,19,20^. Specifically, a prospective study of microdosing that used a self-blinding intervention to approximate placebo control among 191 participants over 4 weeks identified improvements in emotional well-being among microdosers, but noted that correct identification of condition was reported by 72% of participants complicating the ability to conclusively estimate the influence of placebo effects on observed changes. Similarly, a double-blind placebo-control crossover study of 30 respondents followed for eight weeks identified higher levels of self-reported awe in response to aesthetic experiences among microdosers relative to controls. Nonetheless, authors acknowledged the potential confounding effects of breaking blind, as two-thirds of participants accurately guessed their condition^19^. Further analysis of the same participant pool did not identify differences in symptoms of anxiety and depression among microdosers relative to placebo^20^. However, the study noted that participants’ prior experience with psychedelics in addition to low levels of depression and anxiety may have resulted in attenuated microdosing effects. Moreover, a significant proportion of participants correctly guessed their condition in half the trial blocks, however the apparent null effects in the study may render the potential influence of placebo less germane.

Breaking blind and the broader category of expectancy or placebo effects are identified challenges to the interpretation of studies of regular doses of psychedelics^21^, and may also complicate the interpretation of microdosing research. For example, the longitudinal study which attempted to adjust for expectancies by controlling for scores on a modified measure of microdosing expectancies^18^ noted that more than 80% of participants reported prior experience using psychedelics, which makes it likely that scores on the expectancies measure were influenced by past experiences of direct drug effects. Individual differences in drug response due to metabolism and numerous other factors make past pharmacological drug effects likely to be strongly correlated with subsequent direct pharmacological effects, and as such partialing out (i.e. controlling for) expectancies may underestimate direct pharamacological effects^22,23^. In sum, extant longitudinal studies have observed positive effects associated with microdosing but have not been able to confidently estimate the direct pharmacological contributions to such effects. More broadly, parsing direct effects of psychedelics from indirect factors such as set, setting, individual differences, and expectancies presents epistemological and practical challenges, and the study of psychedelics may be best served by going beyond a potentially Procrustean emphasis on blinding and other approaches to maximizing control^24^. For example, the naturalistic examination of large cohorts provides powerful opportunities to examine the consistency of effects across subgroups, and the use of a comparison group absent the premise of blinding, but with similar demographic characteristics and roughly equivalent levels of study-related activities allows for the assessment of the impact of microdosing as distinct from indirect effects such as study engagement, practice effects, regression to the mean, and other potential artefacts common to prospective research.

Concurrent with the increased interest in microdosing psilocybin-containing mushrooms is an acceleration of interest in other putatively therapeutic fungi. In particular, lion's mane, (Hericium erinaceus; HE), has garnered substantial interest for its proposed treatment of depression^25^ and mild cognitive impairment^26^ and preclinical evidence of facilitation of neurogenesis with implications for treating neurodegenerative disorders^27,28^. Recent evidence suggests that some microdosers combine psilocybin with HE in a process referred to as *stacking*^8^. A cross-sectional survey of over 4000 microdosers, which used a sample that partially overlaps with that of the present study, found that over 50% of psilocybin microdosers combined psilocybin with diverse substances, and that HE was the most prevalent addition followed by a combination of niacin (vitamin-B3) and HE^8^. As this is the lone study to report on stacking, the generalizability of these results is unknown. The combination of HE, B3, and psilocybin has been popularized in informal microdosing information networks based on the conjecture that B3 may facilitate psilocybin and HE bioavailability via vasodilation^29^. The salutary effects of both psilocybin and HE have been proposed to operate via BDNF-related processes, raising the possibility of super-additive effects^30,31^. However, the potential effects of psilocybin and HE—with and without B3—have yet to be formally investigated, and the popularity of stacking likely derives from self-experimentation and anecdotal reports.

In sum, despite suggestive results and expanding public interest, the empirical literature remains equivocal on the consequences of microdosing. Further research with control groups and large samples that allow for the examination of potential moderators such as mental health status, age, and gender are required to better appreciate the health consequences of this emerging phenomenon. In the present study, we aim to extend this literature by examining prospective changes associated with microdosing psilocybin as compared to a non-microdosing control group on domains of mental health, mood, and cognitive and psychomotor functioning. To our knowledge, this is the largest prospective study to date of microdosing psilocybin, the first to distinguish between microdosing admixtures (i.e., stacking), and among the few prospective studies to systematically disaggregate analyses according to age and mental health concerns.

## Methods

### Design and participants

We collected longitudinal data between November 2019 and May 2021 from self-selected respondents (Table 1) drawn from a larger study of psychedelic microdosing. The sample partially overlaps with a much larger sample described in a prior cross-sectional study^8^; but adds participants recruited subsequent to that analysis, and excluded participants who did not complete follow-up assessment at one-month and whose microdosing did not include psilocybin. The study materials were integrated within an application available to Apple iOS users^32^ who met the inclusion criteria of being 18 years of age or older, able to read in English, and having access to an iOS device. Both microdosers and individuals not engaging in a microdose practice were eligible for study participation and were simultaneously recruited through media related to psychedelic use and through presentations at psychedelic research and education events.

**Table 1:** Participant characteristics.

	Total (N = 1133)	Microdosers (*n* = 953)	Non-microdosers (*n* = 180)
**Ethnicity**			
White*	85.7% (971)	86.7% (826)	80.6% (145)
LatinX	7.8 (88)	7.3% (70)	10% (18)
Asian	2.6% (30)	2.5% (24)	3.3% (6)
Black	2% (23)	2% (19)	2.2% (4)
**Gender**			
Male	69.7% (788)	69.7% (663)	69.4% (125)
Female	29.4% (333)	29.4% (280)	29.4% (53)
Transgender/non-binary/other	0.9% (10)	0.8% (8)	1.1% (2)
**Sexual Orientation**			
Straight/Heterosexual	88% (991)	88.4% (836)	86.1% (155)
LGBTQ2S+	12% (135)	11.6% (110)	13.9% (25)
**Age**			
18–24**	9.4% (107)	8% (76)	17.2% (31)
25–54**	79.2% (897)	81.5% (777)	66.7% (120)
55+*	11.4% (129)	10.5% (100)	16.1% (29)
**Employment**			
Full-time*	59.5% (668)	60.9% (575)	52.2% (93)
Part-time	13.6% (153)	12.9% (122)	17.4% (31)
Student**	6.9% (77)	5.9% (56)	11.8% (21)
Other	20% (224)	20.2% (191)	18.5% (33)
**Income**			
Under $10,000	4.3% (46)	4% (36)	6% (10)
$10,000-$29.999	16.1% (174)	15.1% (138)	21.4% (36)
$30,000-$89,999	44.7% (482)	45.4% (414)	40.5% (68)
Above $90,000	34.9% (377)	35.5% (323)	32.1% (54)
**Education**			
Graduate degree	18.6% (209)	17.7% (167)	23.3% (42)
Post-Secondary	58.8% (662)	59.6% (563)	55% (99)
Secondary	21.5% (242)	21.7% (205)	20.6% (37)
Less than secondary education	1.1% (12)	1.1% (10)	1.1% (2)
**Community setting**			
Suburban	41.4% (466)	41.7% (394)	40% (72)
Urban	40.7% (458)	40.1% (379)	43.9% (79)
Rural	17.9% (201)	18.2% (172)	16.1% (29)
**Mental health or substance use problems**	28.5% (316)	28.9% (269)	26.3% (47)
**Microdosing practices**			
Dose			
High		10.6% (101)	
Medium		72.6% (692)	
Low		16.8% (160)	
Average monthly microdose days			
21 days or more		12.7% (121)	
11–20 days		53.4% (509)	
Under 10 days		33.9% (323)	
Stacking			
Psilocybin only		40.4% (385)	
Psilocybin & Lion’s Mane		31.9% (304)	
Psilocybin, Lion’s Mane & Niacin		27.7% (264)	

* = *p* < .05, ** = *p* < .01*.* For variables with missing data, percentages reflect proportions of the total valid, non-missing, responses within a category. Multiple category selection was available to participants for ethnicity and stacking. Dose categories for Psilocybin are as follows: Low ≤ .1 g, Medium = 0.1–0.3 g, High ≥ 0.3 g. For supplementary analyses of stacking microdosers were divided into three groups: psilocybin only (n = 385, 40.4%), psilocybin-HE (*n* = 304, 31.9%) and psilocybin-HE-B3 (*n* = 264, 27.7%).

The study consisted of a baseline assessment completed at the study outset, and a follow-up assessment completed 22–35 days later; the assessment schedules were equivalent for both microdosers and non-microdosers. The assessments queried past month psychedelic practices, mood and mental health, and presented tasks testing cognitive and psychomotor processing. Each assessment took 20–30 min to complete with variability due to branching such that many items were only presented to a subset of participants. Informed consent was obtained from all participants, the study was approved by the University of British Columbia Research Ethics Board (H19-03051) and all methods were carried out in accordance with their guidelines and regulations. Hypotheses and outcomes were not pre-registered.

Descriptive data are in Table 1. Age was assessed categorically and dichotomized into *55 and over* and *under 55* to assess potential group differences while considering group sizes to maintain statistical power. The presence of *Mental Health Concerns* was queried with the item *“Do you currently have any psychological, mental health or addiction concerns*?” *Mood* was assessed with the *Positive And Negative Affect Schedule (PANAS)*^33^, a 20-item self-report measure with 10-item subscales that assess positive and negative affect. Due to technical error, one item in the negative subscale was excluded; to address this, scores were converted to percentages. *Depression, anxiety, and stress* were measured with the *Depression Anxiety Stress Scale-21 (DASS-21*)^34^, which has three subscales with 7 items scored on a 4-point scale. *Cognitive and psychomotor tasks* were adapted from the Apple Research Kit, which has been used and validated in several large studies^35,36^. *Psychomotor ability* was measured using an adapted version of the finger tap task^37^ wherein participants tapped two adjacent circles on the screen of an iOS device in an alternating pattern for 10 s using the index and middle finger of their dominant hand. Similar smartphone finger tap protocols have demonstrated good predictive and discriminant validity for neurodegenerative disorders^37,38^. *Spatial memory span* was assessed with an adapted version of the Corsi Block-Tapping task^39^ in which participants recalled the placement of stimuli on a square grid over a time-limited series of rounds of increasing difficulty^40,41^. The criterion used in this study was the number of correct responses. *Processing speed* was assessed with an adapted Paced Auditory Serial Addition Test (PASAT)^42,43^, which involves iterative summing of alternating integers; total correct responses was the criterion.

### Statistical analyses

Mixed linear effects models were generated across 8 outcomes: DASS depression, DASS anxiety, DASS stress, PANAS positive, PANAS negative, finger tap test number of taps, spatial span score, and PASAT score. Multilevel modelling was selected for analysis for its ability to simultaneously test between- and within- group differences, incorporate unequally spaced observations among participants, as was common in the present study, and for its robustness to Type I error inflation resulting from multiple testing^44^. All models included the continuous effect of *Time* (days since baseline response) and the dichotomous *microdose* group factor (*non-microdosers* coded as 0, *microdosers* coded as 1). To build parsimonious models and maintain adequately sized sub-groups, only one additional moderator was included in the models. Age was examined as the moderator for tests of cognitive functioning and mood, whereas given its relevance to the DASS domains of depression, anxiety and stress, the presence of mental health concerns was examined instead of age as the moderator in the three models that examined DASS scores. Specifically, *Age* was entered as a dichotomous between-person variable in models with PANAS; finger tap, PASAT and spatial span tests, and *Mental Health Concerns* was included in models with DASS domains.

Models were built such that variables were retained if they predicted model outcome or were a constituent of a higher level significant interaction. For outcomes where a Microdose*Time interaction was identified, full factorial models were built including the three-way interaction of Microdose*Time*Gender, and either Microdose*Time*Age or Microdose*Time*Mental Health Concerns and all lower level main and two-way interaction effects. Supplementary analyses removed outlier responses that exceeded two standard deviations from the mean of its respective group. A second set of supplementary analyses excluded participants who reported microdosing prior to study initiation to control for potential carry over effects associated with microdosing history. Specifically, participants who reported an active microdosing practice at the baseline assessment were removed; thus we compared microdosers who initiated their practice subsequent to baseline and follow-up assessment to those who did not microdose during this period. To assess stacking, we followed up these analyses with up to three sets of supplementary analyses in the *microdoser* group. These supplementary analyses were limited to outcomes that evinced a Microdose*Time effect in order to prevent inflation of Type I error due to multiple testing. In the first of these analyses, *Psilocybin* + *HE microdosers* were compared to *Psilocybin only microdosers*. A second set of analyses compared *Psilocybin* + *HE* + *B3 microdosers* to *Psilocybin only microdosers*. As in the primary analyses, we also examined moderating effects of either *Age* or *Mental Health Concerns.* In cases where either of these two supplemental analyses noted significant two- or three-way interaction effects, they were followed with a final supplemental analysis that compared *Psilocybin* + *HE* to *Psilocybin* + *HE* + *B3 microdosers*. Chi-squared analyses assessed subgroups for equivalency across age, past-month microdose days, and microdose dosage; differences in these factors were controlled for by randomly trimming participants from the subgroup that was disproportionately high until group proportions were noted as statistically equivalent^45^. Cohen’s *d* was calculated for effects to facilitate interpretation and allow comparison of effect sizes across groups and with past research (Table 2).

**Table 2:** Microdosers versus non-microdosers across one-month.

	Non-microdoser			Microdoser			Between-group one month
	Baseline	One month	*d*	Baseline	One month	*d*	*d*
DASS -Depression*	13.08 (10.39)	11.9 (10.06)	0.1	13.05 (11.22)	8.18 (8.62)	0.49	0.39
DASS – Anxiety*	7.76 (8)	7.83 (7.59)	< 0.01	7.89 (7.42)	5.60 (6.05)	0.34	0.32
DASS – Stress*	15.29 (9.90)	13.39 (9.86)	0.19	15.75 (6.7)	11.04 (8.19)	0.63	0.26
PANAS Positive*	54.69% (15.83)	57.92% (15.38)	0.21	55.07% (15.93)	67.60% (13.79)	0.84	0.66
PANAS Negative*	45.42% (15.84)	42.68% (16.26)	0.17	46.32% (16.31)	36.73% (13.06)	0.65	0.4
Finger Tap*	75.08 (35.61)	79.03 (31.46)	0.1	67.29 (32.76)	78.18 (31.53)	0.34	0.02
PASAT	32.82 (14.32)	39.28 (14.07)	0.45	33.37 (14.08)	39.15 (13.55)	0.42	0.01
Spatial Span	229.39 (63.15)	278.20 (70.24)	0.73	229.78 (53.57)	280.43 (57.35)	0.91	0.03

*Indicates *microdose X time p* < .01.

## Results

*Microdosers* were more likely than *non-microdosers* to be older (*x*^2^ (2, N = 1133) = 22.13, *p* < 0.01), of White ethnicity (*x*^2^ (1, N = 1133) = 4.62, *p* = 0.03) and to report full-time employment (*x*^2^ (3, N = 1122) = 11.83, *p* < 0.01); groups were equivalent in all other demographic domains (all *x*^2^*’s* < 6.03, all *p’s* > 0.05; see Table 1). Comparisons among microdosers of dosage and past-month microdose days found no differences across *Age* (days: *x*^2^ (2, N = 953) = 3.37, *p* = 0.19; dose: *x*^2^ (2, N = 953) = 3.31, *p* = 0.19) and *Mental Health Concerns* (days: *x*^2^ (2, N = 931) = 0.71, *p* = 0.70; dose: *x*^2^ (2, N = 931) = 0.21, *p* = 0.90).

Preliminary analyses identified expected differences according to *Age*; the under 55 group demonstrated superior performance to the 55 + group on all cognitive tasks; for Tap Test (Mean = 70.48 (33.18) versus 52.60 (29.99); *t* (1, 863) = 5.05, *p* < 0.01*);* for PASAT (Mean = 33.67(14.21) versus 30.37 (12.92) *t* (1, 772) = 2.08, *p* < 0.05) and Spatial Span (Mean = 236.25 (51.02) versus 176.88 (58.80); *t* (1, 943) = 11.00, *p* < 0.01). Baseline differences by *Age* were identified for negative mood (mean = 46.89 (16.13) versus 40.64 (16.06); *t* (1, 1048) = 3.96, *p* < 0.01) but not positive mood (mean = 55% (16) versus 55.03% (15.01); *t* (1, 1048) = − 0.018, *p* = 0.99). As expected, participants who reported *Mental Health Concerns* evinced higher scores on all three DASS subscales: Depression (mean = 10.44 (9.72) versus 18.92 (12); *t* (1, 1010) = − 11.81, *p* < 0.01*);* Anxiety (mean = 6.38 (6.36) versus 11.38 (8.74); *t* (1, 1010) = 10.09, *p* < 0.01) and Stress (mean = 13.84 (9.1) versus 20.04 (9.8); *t* (1, 1010) = 9.61, *p* < 0.01). Gender analysis revealed no main effect of gender across time in any of the DASS domains (All F < 1.6, *p* > 0.20).

### Depression, anxiety, stress

**Figure 1:**
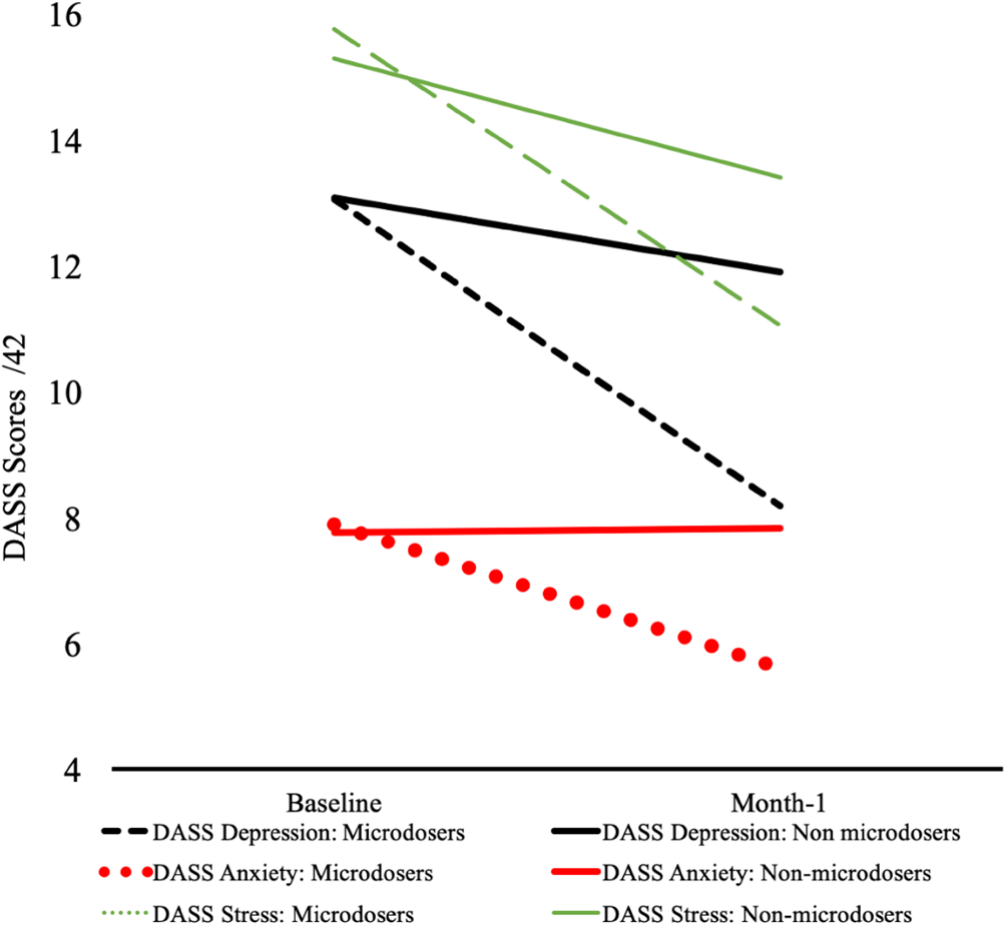
Microdosing and mental health. “Baseline” values reflect the mean participant responses collected 0–7 days from study initiation. “Month-1” values reflect the mean participant responses collected 22–35 days following study initiation.

Comparisons of microdosers to non-microdosers in change from baseline to month-1 (Microdose*time) indicated greater improvements among microdosers across the DASS domains of *Depression* (*F* (1, 1019) = 17.91, *b* = 0*.*12*, p* < 0.01), *Anxiety* (*F* (1, 1017) = 18.33, *b* = 0.08*, p* < 0.01), and *Stress* (*F* (1, 1016) = 15.60, *b* = 0.08*, p* < 0.01) (Fig. 1; Table 2). These effects remained consistent following the removal of 124, 82, and 75 outliers within *Depression, Anxiety,* and *Stress* domains respectively for scores exceeding 2 standard deviations from the mean (all Microdose*time F > 7.99 *p* < 0.01), and in parallel analyses restricted to the 594 participants who did not report microdosing prior to baseline (all Microdose*time F > 4.17, *p* < 0.05). We identified a Microdose*Gender*Time interaction such that the effect of microdosing over time was found to be moderated by gender in DASS *depression.* Specifically, microdose-related reductions in depression were stronger among females than among males (*F* (1, 1016) = 6.61, *b* = 0.17*, p* = 0.01). No Microdose* Gender*Time interaction was identified for DASS *anxiety* (*F* (1, 1024) = 1.14, *b* = 0.46, *p* = 0.29) or DASS *stress* (*F* (1, 1023) = 0.90, *b* = 0.05, *p* = 0.34)*.*

The interactions between *Mental Health Concerns and Microdose* groups were not significant for any of the domains (all Microdose*Mental Health Concerns*Time Fs < 1.16; *p* > 0.10), indicating that the main effects of microdosing were consistent across respondents with and without mental health conditions. Among microdosers with *Mental Health Concerns,* scores on *depression* changed from 18.85 (12.03) at baseline to 11.73 (9.85) at Month-1; for *anxiety,* 11.04 (8.48) at baseline to 7.46 (6.68) at Month-1; and for *stress,* 19.93 (9.71) at baseline to 13.91 (9.02) at Month-1. Among respondents without a history of *Mental Health Concerns,* scores on *depression* changed from 10.40 (9.78) at baseline to 6.65 (7.60) at Month-1; for *anxiety,* 6.53 (6.50) at baseline to 4.81 (5.57) at Month-1; and for *stress,* 13.96 (9.12) at baseline to 9.78 (7.50) at Month-1. Supplementary analyses compared stacking conditions on changes in DASS depression, anxiety and stress scores from baseline to month-1. No differences between *Psilocybin Only Microdosers* and *Psilocybin* + *HE Microdosers (all* F < 0.70; *p* > 0.10) were noted. Likewise, no differences between *Psilocybin Only Microdosers* and *Psilocybin* + *HE* + *B3 Microdosers* were identified *(all* F < 0.77; *p* > 0.10*).*

### Mood

Findings across both PANAS subscales mirrored those of the DASS. Relative to *non-microdosers, microdosers* exhibited greater increases in positive mood from baseline to month-1 (F (1, 1058) = 59.98, *b* = − 0.32, *p* < 0.01) and larger decreases in negative mood over the study duration (F (1, 1059) = 33.76, *b* = 0.23*, p* < 0.01). These effects remained consistent following the removal of 75 and 76 outlier responses within the domains of positive and negative mood respectively for scores that exceeded two standard deviations above or below the mean (all Microdose*time F > 26.32; *p* < 0.01), and among the 479 participants who were microdosing at the time of study initiation (all Microdose*time F > 22.05; *p* < 0.01). Additionally, moderator analyses indicated that these effects remained stable across gender (all Microdose*Gender*Time F < 1.94; *p* > 0.05).

The interaction between age, microdose status and time was not significant for either positive mood (*F* (1, 1058) = 0.21, *b* = − 0.05, *p* = 0.65) or negative mood (*F* (1, 1059) = 1.38, *b* = 0.13 *p* = 0.24), indicating equivalence of mood effects across age. Follow-up analyses did not identify significant differences in changes in either positive or negative mood over time between *Psilocybin Only Microdosers* and either the *Psilocybin* + *HE microdosers* (all F < 0.52, *p* > 0.47) or the *Psilocybin* + *HE* + *B3 microdosers* (all F < 2.44, *p* > 0.12).

### Psychomotor performance and cognition

Analyses of the finger tap test identified a main effect for microdosing, such that *microdosers* demonstrated a more positive change in performance than non-microdosers (F (1, 886) = 9.09, *b* = − 0.24, *p* = 0.03*;* Table 2). Supplementary analyses did not reveal a significant 3-way interaction across *Microdose, Gender and Time,* indicating that microdosing effects were consistent across *Gender* (*F* = 0*.*26*, b* = 0.94, *p* = 0*.*61). The effect of microdosing on tap score over time was robust to the removal of 16 outlier responses with scores 2 standard deviations from the mean (Microdose*Time F = 7*.*23*, b* = − 0.21, *p* = 0.07), and treatment effects remained consistent when the study sample was limited to the 515 participants that were not microdosing at baseline (Microdose*Time F = 5*.*07*, b* = 0.22, *p* = 0.03). Finally, the interaction between Microdose* Time*Age was not significant (*F* = 3*.*41*, b* = 0*.*43*, p* = 0*.*06), indicating that the effect of microdosing was consistent across age.

**Figure 2:**
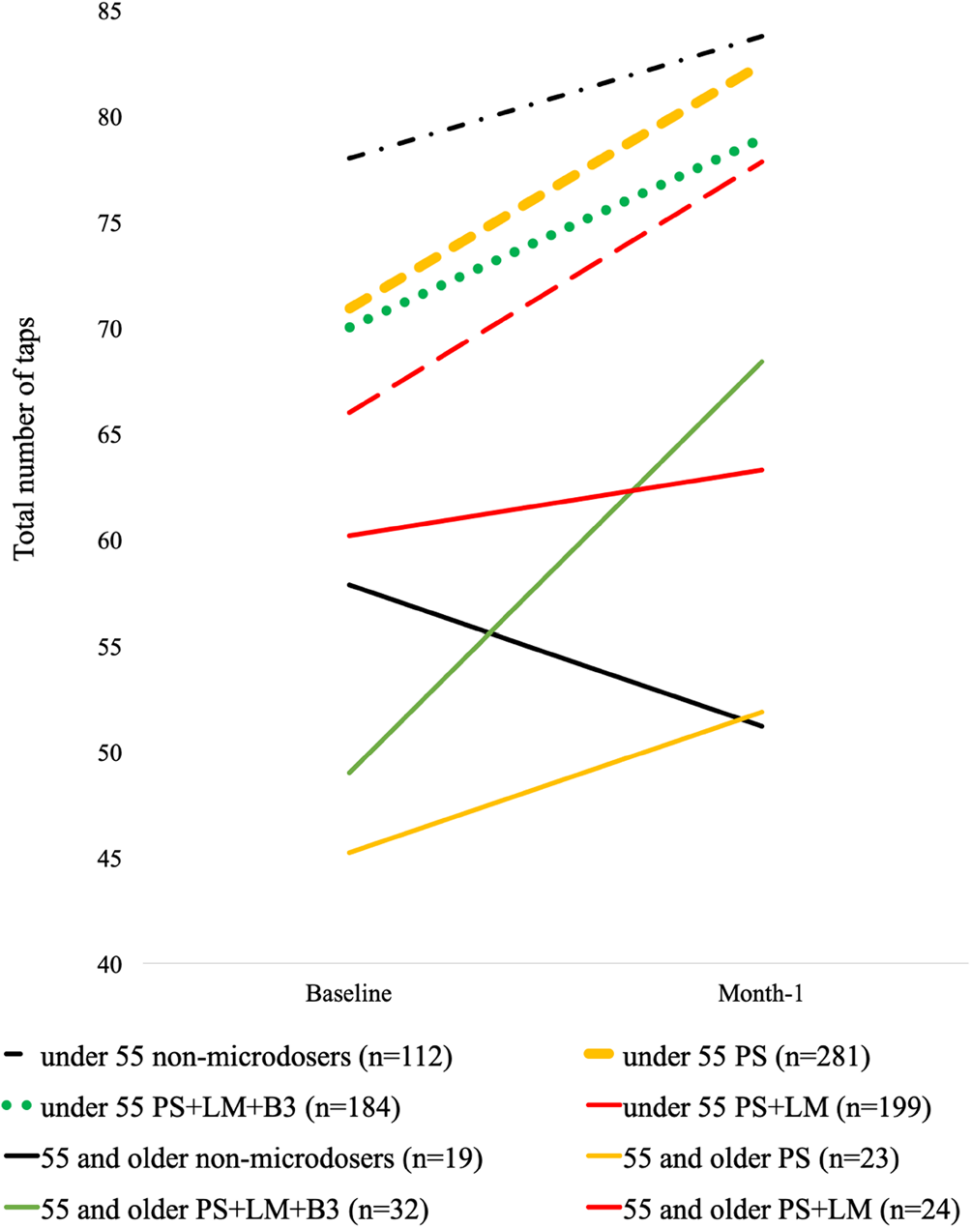
Microdosing and Finger tap test scores**.** “Baseline” values reflect the mean participant responses collected 0–7 days from study initiation. “Month-1” values reflect the mean participant responses collected 22–35 days following study initiation. PS refers to participants who microdosed with psilocybin in the absence of Lion’s Mane (HE). PS + HE refers to participants that microdose with psilocybin and HE in the absence of niacin (B3). PS + HE + B3 microdosers refers to participants who microdose psilocybin with both HE and B3. Differences in group condition slopes were the subject of Microdose*Time interaction analyses.

Analyses of stacking among microdosers (Fig. 2) found no interaction of *Psilocybin only versus psilocybin* + HE*Time, suggesting that the addition of HE did not impact the effect of psilocybin on finger tap (F (1, 524) = 0.284, *b* = 0.12, *p* = 0.67). In contrast, the *Psilocybin only vs psilocybin* + *HE* + B3*Time interaction indicated relatively greater improvement in tap scores with the addition of both HE and B3 to psilocybin (F (1, 732) = 3.93, *b* = − 0.51, *p* < 0*.*05). This finding was followed by examination of the moderating effect of age, which identified a *Psilocybin only vs psilocybin* + *HE* + B3 * Time *Age interaction (F (1, 732) = 8.4, *b* = 0.6*, p* = 0.004), which reflected that the addition of HE and B3 was impactful among older respondents but not among younger respondents. Supplementary analyses of *Psilocybin* + *HE vs psilocybin* + *HE* + B3 * Time revealed a trend toward significance (F (1, 427) = 3.26, *b* = − 0.56*, p* = 0.07), and the three-way *Psilocybin* + *HE vs psilocybin* + *HE* + B3 * Time*Age interaction was identified (F (1, 427) = 6.71, *b* = 0.66*, p* = 0*.*01), indicating that effects were more pronounced among older respondents. Follow-up supplemental analyses indicated that these findings were robust after controlling for subgroup differences in age, microdose frequency and microdose dosage (all 3-way interaction Fs > 6.20, *p* < 0.05).

Comparisons of *microdosers* to *non-microdosers* in change from baseline to month 1 indicated no differences for either the *spatial span task* (F (1, 944) = 0.24, *b* = − 0.07, *p* = 0.63) or the *PASAT* (F (1, 775) = 0.21, *b* = 0.02, *p* = 0.65). In light of this absence of main effects, no follow-up analyses were conducted.

## Discussion

The findings of this study contribute to the growing literature on microdosing in several ways. First, although our study design differs substantially from the designs of the relatively few prior longitudinal studies of microdosing psychedelics—particularly with regard to attempts to account for the potential influence of expectancies—our findings of improved mood and reduced symptoms of depression, anxiety and stress are nonetheless generally similar in direction and size to the unadjusted small to medium positive effects reported in those investigations^7,17,18^. To our knowledge, this is the largest longitudinal study to date of microdosing psilocybin and one of the few studies to engage a control group^17–20^. In light of these methodological strengths, the comparability of our findings with those of prior research from diverse locations and with different methodologies suggests a relatively consistent association between microdosing and improved mental health. Notably, the subgroup of respondents who reported mental health concerns at the time of baseline assessment exhibited an average reduction in depressive symptoms that resulted in a change from moderate to mild depression following approximately 30 days of microdosing psychedelics^46^. Considering the tremendous health costs and ubiquity of depression, as well as the sizable proportion of patients who do not respond to extant treatments, the potential for another approach to addressing this deadly disorder warrants substantial consideration. The potential that psilocybin microdosing may provide a means to improve depression and anxiety clearly points to the need for further research to more firmly establish the nature of the relationship between microdosing, mood and mental health, and the extent to which these effects are directly attributable to psilocybin.

A potential contribution of future research with placebo-controlled designs would be the capacity to disaggregate the contributions of positive expectancies and placebo effects. Although our use of a non-microdosing group that was equivalent to microdosers with regard to demographics and engagement with study procedures is a clear strength, both microdosers and non-microdosers in our study were aware of their status from the onset of the study, making it impossible to rule out the contributions of greater expectancies among the microdosing versus non-microdosing group. However, in consideration of the challenges associated with conducting RCTs in the current restrictive regulatory environment and recognizing the broader challenges to effective blinding of a research drug with well-known and distinctive psychoactive effects, we encourage research to take an expansive perspective on putative placebo effects^24^. Specifically, the clinical reconsideration and study of psychedelics presents an opportunity to reevaluate the extent to which expectancies and frank psychoactive effects might combine to influence subjective well-being in potentially meaningful ways^19^.

The impact of microdosing on tests of cognitive and psychomotor functioning was mixed and limited to psychomotor performance, with no apparent impact on spatial memory or processing speed. Moreover, the magnitude of these effects appeared to be contingent on age and on combining psilocybin with both HE and B3. This specificity for psychomotor performance and reliance on the combination of constituents warrant cautious interpretation, as the literature on microdosing and cognitive performance is scant^18^ and no prior studies have focused on the combination of psilocybin with other putatively active substances. Indeed, although our large sample allowed for a novel level of granularity in our examination of distinct practices among age-related subgroups of microdosers, these groups were nonetheless relatively small, which increases the possibility that our findings of tap test facilitation among individuals over 55 who microdose the combination of psilocybin, HE and B3 may be anomalous. In addition, due to the small number of participants taking B3 without psilocybin or HE, we lacked power to investigate the extent to which these findings were driven by the combination of psilocybin, B3 and HE, as opposed to B3 alone. Moreover, age was collected categorically and the cut-off age of 55 was selected as a convenience based on power; leaving us unable to determine the extent to which the observed effects would be maintained if other cut scores for age were used. As such, replication is required prior to an estimation of potential clinical implications. Nonetheless, should these findings prove robust across diverse samples and investigators, the present results may represent an important first step in the development of novel treatments for prevalent and refractory neurological disorders. Finally, although these findings might be best described as suggestive they nonetheless add preliminary credence to anecdotal reports of benefit from the specific combination of psilocybin, HE and B3^30^.

In addition to small samples in subgroups, observational design, and a generally exploratory approach, interpretation is further limited by potential response bias related to participant self-selection and recruitment through venues that are favorable toward psychedelic use, which may have resulted in overrepresentation in our sample by individuals who respond favorably to microdosing. Further, unavailability of an Android OS version of the application at the time of study limited participation to those with access to Apple devices. This study also did not investigate the influence of dose and dosing practices on outcomes. Future studies with designs that allow for the careful evaluation of the potency, composition and quantity of microdosed materials will be required to refine our understanding of the influence of these key factors. Likewise, adverse effects and interactions with typical antidepressants and anxiolytics were not assessed; such data will be necessary to inform our understanding of microdosing safety and acceptability. In light of these limitations, we encourage future research that employs a more systematic recruitment approach, and designs that assess optimal dosage, best practices and adverse effects associated with psychedelic microdosing.
