# Author Correction: Psilocybin microdosers demonstrate greater observed improvements in mood and mental health at one month relative to non-microdosing controls

**DOI:** 10.1038/s41598-022-17428-0

**Published:** 2022-07-28

**Authors:** Joseph M. Rootman, Maggie Kiraga, Pamela Kryskow, Kalin Harvey, Paul Stamets, Eesmyal Santos-Brault, Kim P. C. Kuypers, Zach Walsh

**Affiliations:** 1 Department of Psychology, University of British Columbia, Kelowna, BC Canada; 2 Quantified Citizen Technologies Inc., Vancouver, BC Canada; 3 Department of Family Medicine, University of British Columbia, Vancouver, BC Canada; 4 Fungi Perfecti, LLC, MycoMedica Life Sciences, Olympia, WA USA; 5 Department of Neuropsychology and Psychopharmacology, Faculty of Psychology and Neuroscience, Maastricht University, Maastricht, The Netherlands

Correction to: *Scientific Reports* 10.1038/s41598-022-14512-3, published online 30 June 2022

The original version of this Article contained an error, where a *p*-value was incorrect in the Results section under the subheading ‘Psychomotor performance and cognition’.

As a result,

“This finding was followed by examination of the moderating effect of age, which identified a *Psilocybin only vs psilocybin* + *HE* + B3 * Time *Age interaction (F (1, 732) = 8.4, *b* = 0.6*, p* = 0*.*04), which reflected that the addition of HE and B3 was impactful among older respondents but not among younger respondents.”

now reads:

“This finding was followed by examination of the moderating effect of age, which identified a *Psilocybin only vs psilocybin* + *HE* + B3 * Time *Age interaction (F (1, 732) = 8.4, *b* = 0.6*, p* = 0*.*004), which reflected that the addition of HE and B3 was impactful among older respondents but not among younger respondents.”

The original Article has been corrected.

